# Gender Inequalities in Citations of Articles Published in High-Impact General Medical Journals: a Cross-Sectional Study

**DOI:** 10.1007/s11606-022-07717-9

**Published:** 2022-07-06

**Authors:** Paul Sebo, Carole Clair

**Affiliations:** 1 University Institute for Primary Care (IuMFE), University of Geneva, Geneva, Switzerland; 2 Center for Primary Care and Public Health, University of Lausanne, Lausanne, Switzerland

**Keywords:** citation, inequality, publication, researcher, woman

## Abstract

**Background:**

Besides the number of publications, the number of citations is another key metric often used to compare researchers with each other. While women researchers tend to have fewer publications than their men colleagues, the data is scarce for the number of citations. We aimed to determine whether there is a gender gap in citations.

**Methods:**

We used *Web of Science* to retrieve the number of citations per year for all research articles and reviews published between January 2015 and December 2019 in fourteen high-impact general medical journals (impact factor > 5). We used *Gender API* to identify the gender of the first/last authors. We compared the results by gender using multivariable negative binomial regressions (adjusting for intra-cluster correlations within journals).

**Results:**

The gender of the first/last author was determined for 13,218/13,350 (99%) and 11,894/12,026 (99%) articles, respectively. The proportion of women among first/last authors was 40% and 29%, respectively. The median number of citations per year was 5 (IQR = 11.3) for women and 6.8 (IQR = 17.8) for men for first authors (IRR = 1.5 [95% CI = 1.3–1.8], *p* value < 0.001), and 6 (IQR = 12.4) and 7.5 (IQR = 17.4) for last authors (IRR = 1.3 [95% CI = 1.2–1.5], p value < 0.001). Articles whose first and last authors were women were the least cited and those whose first and last authors were men were the most cited.

**Conclusion:**

In this cross-sectional study, we found that articles authored by women were cited less often than those authored by men. Further studies are needed to explore the reasons for these gender differences in article citations.

## INTRODUCTION

The difficulties that women face in academic medicine because of their gender alone are still a reality, including in general internal medicine. While women represent nearly half of the physicians in Organisation for Economic Co-operation and Development (OECD) countries,^1^ they remain a minority in senior academic positions.^2–4^

Sociocultural factors likely play an important role in influencing gender differences in work-family balance. Compared to their men counterparts, women researchers generally spend less time at work and more at home, which may be related to personal decisions, but may also reflect persistent cultural norms and stereotypes.^5–8^ Gender-based discrimination may not be as prevalent in academic medicine as it was a few decades ago, but some forms of sexism, which may be conscious or unconscious, are certainly still present and can greatly influence women’s academic trajectories.^7,9–11^ These stereotypes can clearly discourage talented women from pursuing their academic careers.

Several studies showed that women were underrepresented as authors of scientific articles,^4,12–14^ but few articles examined whether there were gender differences in the number of article citations. However, in addition to the number of publications, the *h*-index, which includes the number of citations in its calculation and is defined as the number of publications *h* that were each cited at least *h* times,^15^ is a quantitative measure that is often used to compare researchers with each other. It takes into account both the productivity of researchers (i.e., the number of their publications) and the impact of their research estimated by the number of their citations.^16,17^ Initiatives such as the DORA declaration (https://sfdora.org/resource/halt-the-h-index/) criticize the use of the *h*-index for research assessment because it does not account for qualitative indicators of success, such as teaching, research quality, and collaborations. It is, however, often used as the metric of choice in academia for evaluating researchers for job offers, tenure, promotion, grants, and membership in learned societies.^17–21^ Therefore, both the number of publications and the number of citations (through the *h*-index) could have a notable impact on the career progression of researchers. A possible gender gap in citations could contribute to gender inequalities in senior academic positions.

In a recent study conducted in Switzerland that assessed the productivity of all hospital-based researchers in general internal medicine (*n* = 367), we found that articles whose first author was a woman were on average less frequently cited than articles whose first author was a man (median number of citations: 1 vs 6).^4^ However, this difference was not statistically significant after controlling for various co-factors.

Another recent study that analyzed 5500 publications in general medical journals showed similar results, with articles whose first or last author was a woman being cited significantly less often than other articles (median: 36 vs. 54 citations for first authorship, and 37 vs. 51 citations for last authorship).^22^ However, the authors included only five journals in their study (*New England Journal of Medicine*, *JAMA*, *BMJ*, *Annals of Internal Medicine*, and *JAMA Internal Medicine*), which limits the generalizability of the results. In addition, they used *Genderize*, a gender-tool that is less efficient than others (e.g., *Gender API* or *NamSor*) in terms of inference accuracy,^23^ and did not perform multivariable analyses.

In the current cross-sectional study, we aimed to determine whether there is a gender gap in citations of articles published in a selection of fourteen high-impact general medical journals.

## METHODS

Based on the *Journal Citation Reports* (JCR) list for the categories “general internal medicine” and “primary health care,” we selected all general medical journals with an impact factor for 2020 greater than five. Then, we used the *Web of Science* (WoS) resources to retrieve all research articles and reviews published between January 2015 and December 2019. We recorded the name of their authors, and the number of citations and citations per year as of 15 December 2021. The data collection was done with the International Standard Serial Number (ISSN) of the journals because journals can have multiple names in WoS. Finally, we used *Gender API* to determine the gender of the first and last author of the selected articles. *Gender API* (https://gender-api.com) is a web-based gender detection tool that infers gender from individuals’ first names. This tool has the great advantage that it can be used even by researchers with little computer knowledge. Its use is indeed extremely simple, since it only requires the uploading of a database in Excel or CSV format (https://gender-api.com/en/excel-and-csv). After processing the data, a “gender” column is added to the initial file. This study complies with the Strengthening the Reporting of Observational Studies in Epidemiology (STROBE) guideline for cross-sectional studies.

We summarized the data by calculating both the mean (SD) and median (IQR) number of citations and citations per year, stratified by journal and gender. We compared the results by gender using Wilcoxon rank sum test (right-skewed citation distribution) and univariable negative binomial regression (count data with over-dispersion). We also performed multivariable negative binomial regressions, adjusting for year of publication (for citations) and intra-cluster correlations within journals (for citations and citations per year﻿). Finally, we examined the data for gender combinations of first and last authors (women-women, women-men, men-women, men-men) and tested for linear trend across these groups using orthogonal polynomial contrasts.

We repeated all analyses with two additional samples consisting of names whose gender could be determined with ≥ 60% and ≥ 80% accuracy, respectively, to exclude ambiguous names that could skew the gender distribution. Indeed, *Gender API* provides an additional parameter that estimates the accuracy of the inference (min 0, max 100%). All names whose gender was estimated with an accuracy of < 60% and < 80%, respectively, were considered non-classifications and therefore excluded from the analysis.

The statistical significance was set at a two-sided *p* value of ≤ 0.05. All analyses were performed with STATA 15.1.

## RESULTS

Fourteen general medical journals were included in the study (Table 1). Impact factors ranged from 91.3 (*New England Journal of Medicine*) to 5.0 (*American Journal of Medicine*). Of the 14,256 articles retrieved by WoS, 13,350 had first names of authors and 1324 had only one author. *Gender API* determined the gender of the first and last author for 13,218/13,350 (99%) and 11,894/12,026 (99%) articles, respectively. The proportion of women among first and last authors was 40% and 29%, respectively, and varied by journal from 27 to 54% and from 20 to 41%.

**Table 1:** Proportion of Articles with Women as First and Last Authors Published in Fourteen High-Impact General Medical Journals, and Number of Citations, Stratified by Gender of First and Last Authors

Journal	2020 JCR impact factor	Number of articles	Proportion of articles first authored by women	Median number of citations (IQR) for articles first authored by women	Median number of citations (IQR) for articles first authored by men	*p* value^*^	Number of articles	Proportion of articles last authored by women	Median number of citations (IQR) for articles last authored by women	Median number of citations (IQR) for articles last authored by men	*p* value^*^
Overall		13218	40.3	23 (56)	31 (90)	< 0.001	11894	29.3	26 (60)	35 (89)	< 0.001
*New England Journal of Medicine*	91.3	1103	26.8	121.5 (222)	161 (264)	0.01	1005	20.7	140.5 (287.5)	165 (272)	0.06
*Lancet*	79.4	1687	32.0	88.5 (183.5)	131 (218)	< 0.001	1605	23.4	106 (181.5)	127 (219)	0.01
*JAMA*	56.3	1086	39.2	70 (118)	96 (144)	< 0.001	995	26.6	85 (120)	97.5 (141)	0.14
*BMJ*	39.9	1308	43.5	24 (63)	34 (68)	< 0.001	990	31.5	41.5 (57.5)	48 (68)	0.11
*Annals of Internal Medicine*	25.4	1199	39.1	8 (43)	4 (45)	0.74	758	27.4	38 (65.5)	30 (59)	0.02
*JAMA Internal Medicine*	21.9	649	41.8	44 (57)	49.5 (58)	0.75	646	32.0	44 (58)	47 (59)	0.42
*PLOS Medicine*	11.1	899	45.1	28 (38)	28 (40)	0.62	898	33.9	25 (39.5)	29 (39)	0.03
*BMC Medicine*	8.8	918	44.9	24 (29.5)	22.5 (32)	0.18	903	30.1	25 (29.5)	23 (33)	0.68
*JAMA Network Open*	8.5	981	44.4	12 (16)	14 (19)	0.02	980	32.8	12 (16)	14 (19)	0.14
*Canadian Medical Association Journal* (*CMAJ*)	8.3	409	44.3	13 (24)	14 (24.5)	0.46	358	29.6	15 (21)	16.5 (26)	0.77
*British Journal of General Practice*	5.4	627	54.4	8 (13)	5 (14)	< 0.001	494	36.8	9.5 (15)	10 (13)	0.95
*Annals of Family Medicine*	5.2	332	51.8	15 (17.5)	14 (14)	0.57	316	36.1	13 (14)	16 (18)	0.12
*Journal of General Internal Medicine*	5.1	1044	50.5	12 (21)	11 (17)	0.04	1035	41.3	11 (17)	12 (19)	0.61
*American Journal of Medicine*	5.0	976	29.8	11 (19)	12 (20)	0.34	911	19.5	9 (16)	13 (20)	< 0.001

^*^Wilcoxon rank-sum test

The median number of citations was statistically higher for articles whose first author was a man vs. a woman in five journals including the four with the highest impact factor (Table 1). In contrast, women’s papers were on average more frequently cited than men’s papers in two journals. For the last authors, the median number of citations was statistically higher for men in three journals and for women in one journal.

When analyzing the data overall (Table 2), the unadjusted and adjusted differences in the number of citations and citations per year between women and men were statistically significant for both first and last authors (all *p* values < 0.001). For example, for first authors, the median number of citations was 31 (IQR = 90) for men and 23 (IQR = 56) for women (adjusted incident rate ratio (IRR) = 1.5 [95% CI = 1.3–1.8], *p* value < 0.001).

**Table 2:** Unadjusted and Adjusted Associations Between Number of Citations and Number of Citations Per Year for Articles Published in Fourteen High-Impact General Medical Journals, and Male Gender

Variable	Number of articles	Number of citations Mean (SD)	Number of citations Median (IQR)	Unadjusted incident rate ratio (IRR)^*,†^	Adjusted incident rate ratio (IRR)^*, ‡^
Citations of articles by gender of first author					
Woman	5324	68.7 (177.9)	23 (56)	1	1
Man	7894	104.1 (255.6)	31 (90)	1.5 (1.4–1.6)	1.5 (1.3–1.8)
Citations by year of articles by gender of first author					
Woman	5324	13.5 (31.0)	5 (11.3)	1	1
Man	7894	20.5 (47.5)	6.8 (17.8)	1.5 (1.5–1.6)	1.5 (1.3–1.8)
Citations of articles by gender of last author					
Woman	3480	77.2 (190.5)	26 (60)	1	1
Man	8414	106.3 (249.2)	35 (89)	1.4 (1.3–1.5)	1.3 (1.1–1.5)
Citations by year of articles by gender of last author					
Woman	3480	15.7 (36.8)	6 (12.4)	1	1
Man	8414	20.7 (45.7)	7.5 (17.4)	1.3 (1.3–1.4)	1.3 (1.2–1.5)
Citations of articles by gender of first and last author				§	‖
Woman first and last author	1726	60.5 (128.7)	24.5 (50)	1	1
Woman first author and man last author	3006	83.4 (207.6)	28 (64)	1.4 (1.3–1.5)	1.3 (1.1–1.4)
Man first author and woman last author	1710	91.4 (229.1)	28 (72)	1.5 (1.4–1.7)	1.5 (1.2–1.8)
Man first and last author	5290	119.2 (270.3)	39 (103)	2.0 (1.8–2.1)	1.9 (1.4–2.4)
Citations per year of articles by gender of first and last author				¶	#
Woman first and last author	1726	12.3 (23.5)	5.5 (10.4)	1	1
Woman first author and man last author	3006	16.1 (35.4)	6.2 (12.8)	1.3 (1.2–1.4)	1.3 (1.2–1.5)
Man first author and woman last author	1710	18.4 (42.8)	6.4 (15.2)	1.5 (1.4–1.6)	1.5 (1.2–1.8)
Man first and last author	5290	23.3 (50.5)	8.3 (20.2)	1.9 (1.8–2.0)	1.9 (1.4–2.5)

^*^All *p* values < 0.001

^†^Univariable negative binomial regression

^‡^Multivariable negative binomial regression (model adjusted for year and journal (citations) and for journal (Citations per year))

^§^*p* value for linear trend < 0.001 (IRR per unit increase in gender group: 1.2 [95% CI 1.2–1.3])

^‖^*p* value for linear trend < 0.001 (IRR per unit increase in gender group: 1.2 [95% CI 1.1–1.3])

^¶^*p* value for linear trend < 0.001 (IRR per unit increase in gender group: 1.2 [95% CI 1.2–1.3])

^#^*p* value for linear trend < 0.001 (IRR per unit increase in gender group: 1.2 [95% CI 1.1–1.3])

The results were all similar for the sensitivity analyses. For example, for the same indicator (i.e., median number of citations for first authorship), retaining only publications for which gender was determined with 60% and 80% accuracy, the adjusted IRR was 1.5 [(95% CI = 1.3–1.8), *p* value < 0.001] and 1.6 [(95% CI = 1.3–2.0), *p* value < 0.001], respectively.

Finally, when analyzing the first/last author combinations (Table 2), there was a linear trend across the groups, with articles with women as first and last authors being cited least often and articles with men as first and last authors being cited most often.

## DISCUSSION

In this cross-sectional study, we found that articles published in high-impact general medical journals were on average cited less often when first/last authors were women vs. men. Articles whose both first and last authors were women were cited the least often.

### Comparison with Existing Literature

Our results confirm those of the recent study conducted by Chatterjee and Werner on 5500 articles published in high-impact general medical journals (median number of citations: 36 for women vs. 54 for men for first authorship, and 37 vs. 51, respectively, for last authorship).^22^ In a previous study assessing the productivity of Swiss researchers in general internal medicine, we found that the gender difference in the number of citations per publication (median number of citations: one for women vs six for men) was not statistically significant in multivariable analysis.^4^ This lack of significance was probably related to the small sample size (N = 367 researchers).

Several hypotheses can in our opinion be considered to explain the gender gap in citations. Compared to men, women may be more restrained in the way they promote their research^24^ and are less often invited to medical conferences to present their studies.^25–27^ Their research may therefore be less known to the scientific community. It is also possible that women and men researchers differ in the research topics they address and how they address them.^28,29^ Since the majority of studies are conducted by men, these studies may be more likely to cite other articles authored by men. Because of the balance between career and family aspirations that is socially imposed on women, women researchers often have fewer years of practice behind them, and thus less experience, than their men colleagues of the same age. In addition, women’s research is not as well funded as men’s. With less experience or research projects that receive less funding, it can be hypothesized that women choose research topics or study designs that are considered as less “prestigious” and less valued by the scientific community. The proportion of researchers with an academic affiliation or with a particular type of affiliation is not necessarily the same for women and men. However, depending on the type of affiliation, researchers may have more or less time available to conduct their research. More time may often mean more ambitious studies and therefore of greater potential interest to the scientific community. Finally, men authors may be more inclined to practice self-citation. This technique is a known and opportunistic way to increase the *h*-index. There are probably still other reasons for these gender-related citation differences, which would certainly require further study to investigate in depth.

We also found that, overall, the proportion of women among first and last authors was 40% and 29% respectively. These results are similar to those from other recent studies conducted with general medical journals. For example, in a study examining 44,000 articles published between 2016 and 2020 in the 100 general medical journals with the highest impact factor, we found that the proportion of women among first authors was 41%.^13^ In another study, Hart and Perlis found that women were first and last authors of 42% and 32%, respectively, of research articles published in 2017 in a selection of 15 high-impact general medical journals (2016 impact factors ranging from 17.2 to 4.2).^12^

Various measures may be taken to reduce the gender gap in citations and, more generally, gender inequalities in research. Academic organizations should promote and support women researchers throughout their academic careers, for example, by allowing them to free up time for research, by advocating for gender equity in grants, or by improving the visibility of research done by women (media coverage, communication, conferences). In addition, the way in which research is evaluated should probably be questioned. The *h*-index only partially reflects the quality of research or the involvement of academics. As researchers, we should value the scientific content of studies more than measures such as the *h*-index.

### Limitations

Our study has a large sample size but has several limitations. We were unable to examine the influence of a number of potentially confounding factors, such as authors’ institutional affiliation and career stage, because these variables were not available. Yet, these variables could explain to some extent the observed differences between the number of article citations and gender. In addition, the determination of the gender of the authors was done with a gender detection tool and not by manual internet search. However, the tool used (*Gender API*) has been shown to be accurate,^23^ only 1% of the queries resulted in a non-classification (i.e., undetermined gender), and the results of sensitivity analyses were similar. Finally, using a tool that determines gender on the basis of first names raises ethical considerations, as this method did not allow us to assess non-binary or transgender identity.

## CONCLUSION

In this cross-sectional study that examined all research articles and reviews published between January 2015 and December 2019 in sixteen high-impact general medical journals (i.e., with an impact factor greater than five), we found that publications authored by women were cited less often than those authored by men. We also found that publications with women as first and last authors were the least frequently cited, whereas those with men as first and last authors were the most frequently cited.

Further studies with not only quantitative but also qualitative (or mixed) methods would be needed in the future to confirm our hypotheses regarding the reasons behind gender differences in article citations. The results of these studies could also be useful for implementing measures to ensure that publications by women and men researchers are cited more equitably.

## Data Availability

The data associated with this article are available in the Open Science Framework (DOI 10.17605/OSF.IO/63QNS).
